# The perspective of soft‐robotics for people with neurodegenerative diseases: Can we develop robotic solutions using soft material?

**DOI:** 10.1002/alz.089703

**Published:** 2025-01-09

**Authors:** Bijetri Biswas, Elizabeth Coulthard, Anne Roudaut

**Affiliations:** ^1^ Fairlie Healthcare, London United Kingdom; ^2^ North Bristol NHS Trust, Southmead Hospital, Bristol United Kingdom; ^3^ University of Bristol, Bristol United Kingdom; ^4^ Bristol Medical School, University of Bristol, Bristol United Kingdom; ^5^ North Bristol NHS Trust, Bristol United Kingdom

## Abstract

**Background:**

We investigate perceptions of soft robotics in individuals with neurodegenerative diseases (NDD) from diverse communities. Soft robotics is made from soft, flexible materials to make it safer for users. It is a fast‐emerging medical field with applications ranging from diagnosis to rehabilitation practices. However, this area is underinvestigated in the older population with neurological disorders.

**Method:**

We recruited individuals with NDD, e.g. dementia, Parkinson’s and mild cognitive impairment, via Bristol Brain Centre, charities, personal contacts, and social media advertisements. We aim to understand better user requirements and their views on such innovative technologies. In this ongoing study, we interviewed individuals with NDD, careers and clinicians to find the gap in designing soft robotics and conducted co‐design sessions with each group. To conclude the study, we will collect all the designs, create a prototype and gather iterative feedback.

**Result:**

Preliminary results from the semi‐structured interviews have shown that interaction with soft robotics technologies, such as smart pillows, prosthetics, and others, needs further exploration but is challenging. Therefore, individuals found it difficult to think or acknowledge what could help them improve their quality of life. It also highlights the importance of technologies that can help carers facilitate patients' needs. Interviews with clinicians also emphasised carers' needs and technology that can promote independence and improve well‐being. Another interesting finding from the study is that previously, individuals were unaware of creating technologies personalised to them. Furthermore, co‐designing with this population showed that involving them in the ideation and designing process is critical for empowerment.

**Conclusion:**

Do‐it‐yourself (DIY) practice offers a higher engagement in developing complex technologies, and the user‐centric design process using the Arduino prototyping platform improves the engagement rate. Cultural and ethnic diversity in the study population was another challenge, as it was challenging to get a clear view of their needs due to the language barrier. However, they overcame this by expressing their ideas by drawing. We aim to understand user requirements better and their views on such innovative technologies, present developments in soft robotics technology, and research challenges associated with our population to highlight research directions to understand this emerging field better.

